# Reconstructing strontium-90 intake in beagles using neural networks: a data-driven assessment of historical inhalation records

**DOI:** 10.1088/1361-6498/ae0e7f

**Published:** 2025-11-06

**Authors:** David Carpio Gonzalez, Alexander D Glasco, Gayle E Woloschak, Shaheen Azim Dewji

**Affiliations:** 1 Nuclear and Radiological Engineering and Medical Physics Programs, George W. Woodruff School of Mechanical Engineering, Georgia Institute of Technology, Atlanta, GA, United States of America; 2 Department of Radiation Oncology, Feinberg School of Medicine, Northwestern University, Chicago, IL, United States of America

**Keywords:** internal dosimetry, artificial intelligence, machine learning, dose reconstruction, bioassay, ITRI, beagle dogs

## Abstract

Dose estimation in response to internal radionuclide exposures requires reconstruction of the initial intake activity, which is frequently unknown due to the absence of *a priori* data. In such scenarios, intake is inferred from bioassay measurements obtained at one or more time points post-exposure. Reconstructing an initial intake from bioassay relies on biokinetic models that describe the body distribution and clearance of the toxicant. These models typically employ first-order differential equations with generalised population parameters, which do not capture individual variation in metabolism or anatomy. Thus, reconstruction of initial intakes is affected by multiple sources of stochasticity, including physical deposition of the inhaled radionuclide, detection system uncertainty, and inter-individual physiological variability. The capacity of machine learning (ML) algorithms to model highly non-linear and often stochastic processes makes them appropriate for augmenting intake reconstruction. This study applies artificial neural networks to estimate the initial intake activity of ^90^Sr inhaled by beagles. Model performance and sensitivity to input data quality were assessed through inclusion of individual-specific features, such as age, weight, and sex. Three data regimens were systematically designed, each with distinct pre-processing pipelines and model complexity. The first regimen demonstrates feasibility of intake reconstruction using bioassay measurements taken within 14 days post-exposure. The second regimen demonstrates that summary statistics of retention functions in historical records lack sufficient resolution for individualised ML modelling. The third regimen shows that historical dose estimates, despite limitations in resolution and methodology, can be used as surrogate features when multiple post-exposure time points are available. Root mean squared error was used to evaluate prediction error, while a custom metric, the variance relative difference, quantified model bias. In addition to evaluating predictive performance, this study assesses the integrity and usability of historical data from ^90^Sr beagle inhalation experiments conducted at the Inhalation Toxicology Research Institute between 1966 and 1987.

## 1. Introduction

Internal dosimetry is a multifaceted field in radiation protection associated with the intake of radionuclides, the retention of activity in the body, and the time-varying deposition of energy in different regions of interest within the body. The field relies on computer-assisted analysis, tools and methodologies to achieve the highest accuracy and fidelity of the models. Computational internal dosimetry operates at the intersection of biokinetic modeling, primarily outlined and published by the International Commission on Radiological Protection (ICRP) [1–3], incorporating experimental data acquired from an extensive history of radiobiological and epidemiological studies [4–6], and numerical methods of evaluation and prediction from the biokinetic models [7]. In delineating between acute and chronic intakes, chronic intakes are pervasively studied in occupational hazard and risk assessment applications. Acute intakes, however, while also studied as an occupational hazard, becomes most relevant when considering potential radiological accidents, emergency response, or the dispersal of radioactive material in the aftermath of a nuclear weapon detonation, especially due to the need for mass triage strategies for dosimetry following an acute exposure to a large population.

Beginning in the early 1950s, government-funded large-scale beagle studies were conducted at research sites across the United States to ascertain the health effects of radionuclide internalisation. Several of these studies were conducted at the Inhalation Toxicology Research Institute (ITRI) established by the Lovelace Foundation for Medical Education and Research and funded by the Department of Energy [8]. These studies focused on metabolic, toxicologic, and dosimetric studies in inhalation of fallout-relevant radionuclides [9]. The study conducted herein utilizes data from the ITRI experiments in which beagles were exposed to a relatively soluble form of strontium-90 ($ ^{90}\text{Sr}$ in the form of $ ^{90}\text{SrCl}_2$) and applies deep learning architectures from the available data features. The objective was to assess whether machine learning (ML) methods can reliably estimate the intake activity from acute inhalation exposures based on post-exposure bioassay measurements and individual-specific physiological characteristics (i.e. age, sex, and weight).

Machine learning (ML) is a subset of artificial intelligence (AI) that leverages advanced statistical methods and optimisation methodologies to uncover complex, and often non-linear, relationships in large datasets. Applications frequently involve regression or classification tasks. In the field of internal dosimetry, a persistent scale mismatch between historical and modern animal studies and human datasets limits the ability to draw meaningful conclusions about human internal exposures. To address this limitation, this study provides an in-depth review of an available dataset and its quality. The study thusly evaluates the suitability of the selected data for deep learning applications. The plurality of models implemented demonstrates how deep learning provides a valuable investigative framework for addressing unresolved questions in internal dosimetry, such as the temporal resolution required of bioassay data for reliable reconstruction of intake activity, and whether the quality of historical data is sufficient to train large models or if the data requires extensive post-processing, including recalculation of absorbed dose estimates using modern techniques.

## 2. Background

The final tabulations of the soluble ^90^Sr data were obtained from the ITRI Annual Report LMF-120 (1986–1987) [10] via the Northwestern Radiobiology Archive (NURA) website [11]. Detailed descriptions of the experimental design and methodology are contained in annual reports as early as LF-33 (1966–1967) [12] when the study began. Animals in this study were subject to several routine tests and analyses, including hematologic analysis, physical examinations, microbiological testing and pathological analyses of biopsy and necropsy tissue. This study will focus on the internal dosimetry, including the experimental methods yielding activity measurements used in retention models and dosimetric estimation methods.

The various ITRI studies of radionuclide inhalation shared a common experimental design with key variants being the radionuclide of interest, the solubility, and the age at initial exposure. The initial burdens varied between experimental groups and retained activity was measured at multiple time points post-exposure. The most complete descriptions of the experimental procedures, results, and tabulations of the Beagle Dog experiments are provided in the annual reports of the ITRI, which are identified by series LF or LMF, where the LMF replaced the LF tag starting with Annual Report 76 [11, 13]. An extensive summary of all beagle experiments carried out among several institutions (University of Utah Radiobiology Laboratory, University of California-Davis, Argonne National Laboratory, Pacific Northwest Laboratory, Colorado State University, and the Inhalation Toxicology Research Institute) over four decades is available through the NURA website [11] and from *Life-Span Effects of Ionising Radiation in the Beagle Dog* by Roy C. Thompson of Pacific Northwest Laboratory [8]; a PDF version of the book is also accessible from the NURA website. Thompson’s summary includes all modes of experimentation, including external exposures and insoluble chemical forms of the radionuclides. The current study discusses and employs data from acute inhalation exposures of a soluble form of $ ^{90}\text{Sr}$, as those are most metabolically relevant when attempting to characterise the uncertainty of the biokinetic models and reconstruction of the initial intake.

**Figure 1.**
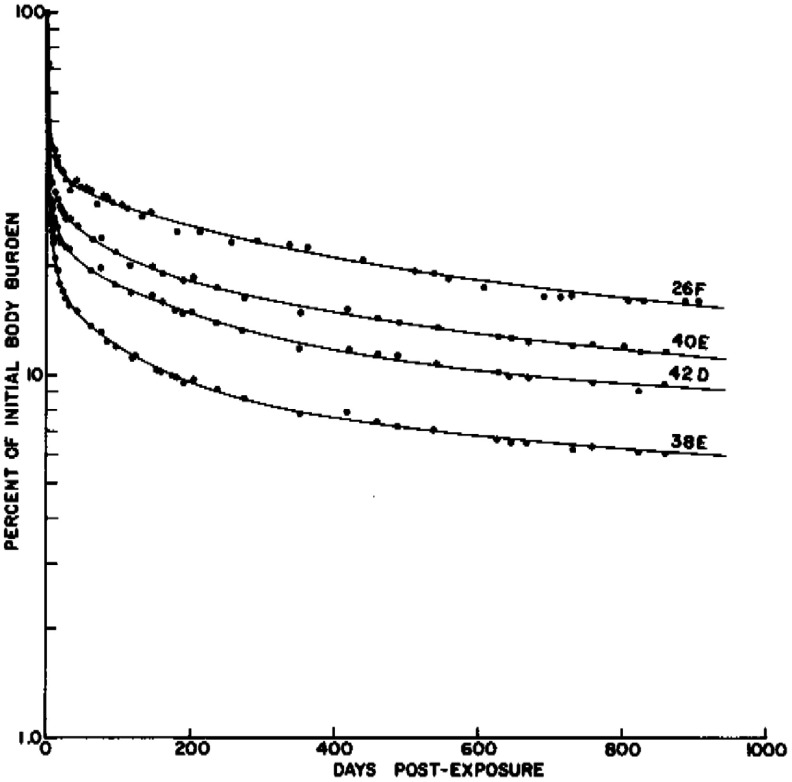
Example of historical retention equations for four selected dogs [14].

The ITRI Annual Report LF-39 documents four experimental groups of 12 dogs each with varying mean initial body burdens, in addition to a cohort of 15 control group dogs [14]. In this experiment, beagles were exposed to strontium chloride in a caesium chloride vector aerosol using a canine inhalation exposure apparatus [15]. The particle size distribution was described by a log-normal function with an activity median aerodynamic diameter (AMAD) ranging from 1.4 to 2.7 micron with a geometric standard deviation of 2 micron. After exposure to achieve a desired initial whole body burden (IBB), each dog was transferred to a whole-body counting facility. After a whole-body counting calibration, the average IBB for each of the four experimental groups were 4.1, 23, 100, and 230 *µ*Ci kg^−1^ [14], which represent the first bioassay of activity available in the dataset. These calculated burdens were derived from repeated whole-body counts in the first hour after exposure to maximise reproducibility. The decline in burden became parallel across dogs following an initial period of rapid clearance, which was characterised by high inter-individual variability. Examples of the retention equations for four individual dogs from the ITRI annual reports are plotted in figure 1, which is the result of a non-linear regression performed for each dog. The experimental protocol associated with figure 1, which plots the data points for each dog’s retention equation, implies the existence of individual whole-body counting measurements; however, these measurements are not present in the historical documentation.

**Figure 2.**
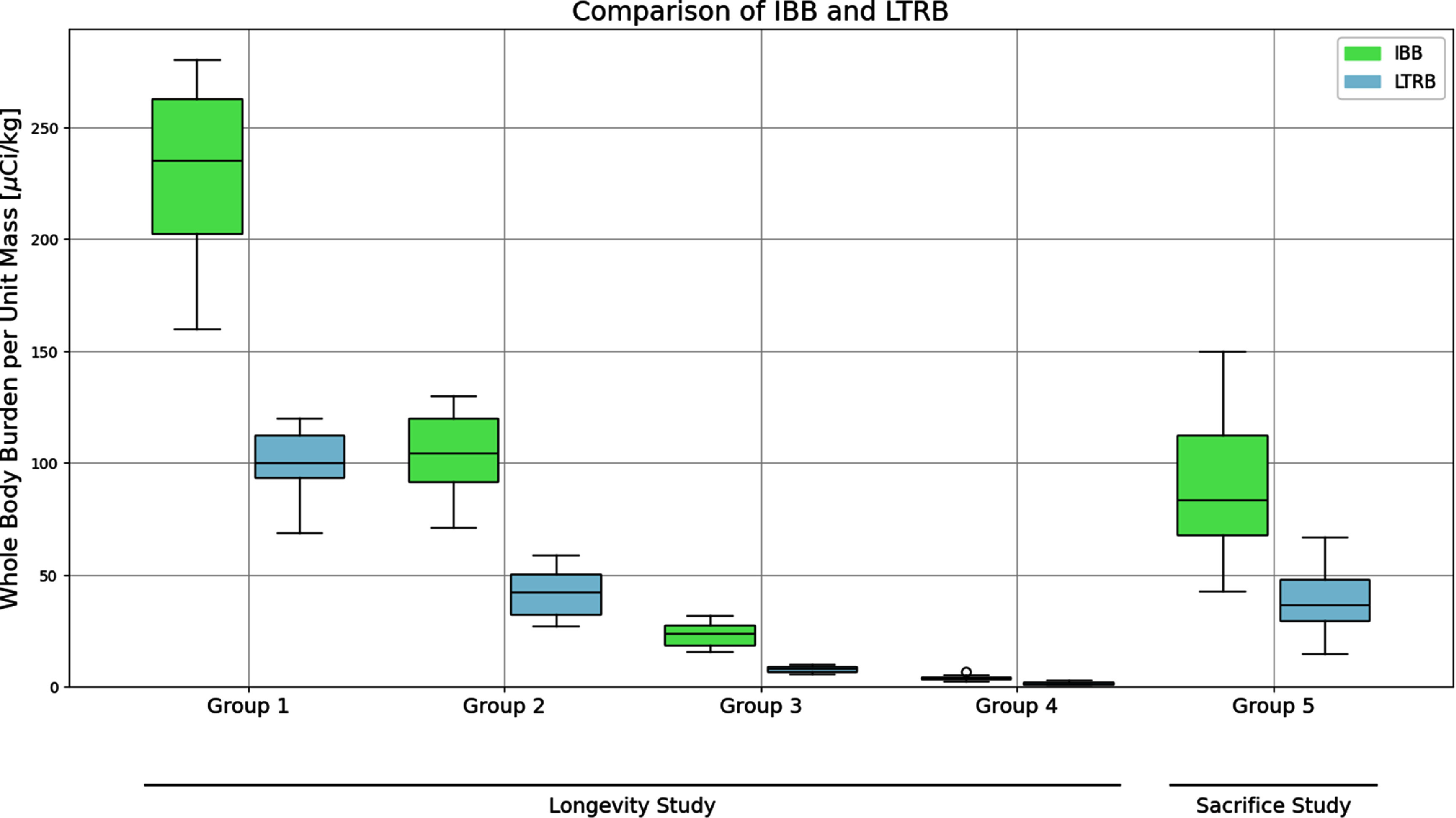
Paired boxplots of IBB and LTRB for the six experimental groups in table 1. All values are positive with a minimum of 1 Ci/kg LTRB occurring in Longevity study experimental group 4. Longevity and Sacrifice control groups are omitted from the plot due to not being exposed (i.e., IBBs and LTRBs are 0)

Fourteen days post-exposure, the dogs were once again subjected to whole-body counting to determine the long term retained burden (LTRB). The mean values for the four dose groups were reported as 1.4, 6.2, 30, and 70 *µ*Ci kg^−1^, respectively. The four dose groups of 12 dogs each and the control group of 15 dogs comprised an overall study group entitled the ‘Longevity Dogs’. In addition, there was a corresponding ‘Sacrificed Dogs’ study that included 24 exposed dogs with a mean IBB of 88.75 *µ*Ci kg^−1^ and LTRB of 37.8 *µ*Ci kg^−1^. These dogs were serially sacrificed as part of the histopathological and radiobiological studies, but their reported data are still included with this study. The summary statistics calculated for this study are shown in table 1. Figure 2 shows the paired boxplots for the IBBs and LTRBs for each of the six experimental groups of table 1, where Groups 1–4 correspond to the longevity study and Group 5 corresponds to the sacrifice study.

**Table 1.** Initial body burdens and long-term retained burdens of the five experimental groups and two control groups.

Experimental Group IBBs and LTRBs							
		IBB (*µ*Ci kg^−1^)			LTRB (*µ*Ci kg^−1^)		
Experimental Group	Number of Dogs	Mean	SD	%RSD	Mean	SD	%RSD
Longevity	12	226.67	40.28	17.77%	99.25	16.41	16.54%
Longevity	12	103.5	17.01	16.44%	42	10.36	24.67%
Longevity	12	23.42	5.42	23.16%	7.83	1.21	15.49%
Longevity	12	4.03	1.08	26.79%	1.75	0.6	34.01%
Longevity	15	0	0	0%	0	0	0%
Sacrifice	24	88.75	29.07	32.07%	37.82	13.74	36.32%
Sacrifice	10	0	0	0%	0	0	0%

## 3. Methodology

The capabilities of ML in resolving highly non-linear systems lend well to the high degree of uncertainty that challenges the field of internal dosimetry [16, 17]. The decision to implement an ML framework for any application requires a data-driven approach that is justified by uncertainty characterised by statistical patterns that must be uncovered in a non-linear formulation. The justification for implementing neural networks in conjunction with the historical beagle dog data is to assess whether a Bayesian inference framework embodied by deep learning models aids in the characterisation of biokinetic uncertainty observed in historical experiments. Furthermore, ML has been discussed as a potential method for leveraging existing datasets, particularly for drawing new conclusions regarding radiation risks [18], or in the case of the present study, for the reconstruction of intake activity [19]. Deep learning frameworks have specifically been outlined as a promising method for solving stochastic biological systems [20], especially when they are informed through physics-based loss functions. Alternatively, neural networks are commonly applied to biomedical data analysis without the inclusion of physics-based loss functions, whether the data are derived from images or biomarkers [21]; the current study is most akin to the latter case, in which the bioassay measurements taken at multiple points in time constitute a representation of the biological system, but the loss functions implemented account solely for the stochastic nature of the system.

Regression-based artificial neural networks (ANNs) designed to reconstruct the activity of an acute radionuclide intake were developed and compared. The models employed various input features, including age, sex, weight, time post-exposure, and measured activity from bioassay to improve predictive accuracy. The underlying premise is that the feasibility of reconstructing acute intake activity using ML for animal datasets suggests feasibility for human internal exposures, provided that the necessary data are available. The network was implemented in a variety of architectures and complexities along with different data preprocessing or generation techniques to cast a wide array of reconstruction configurations built from the ground-up. Furthermore, this study adopts an extension to the traditional ANN architecture to incorporate a Bayesian approach that encompasses both epistemic and aleatoric uncertainty in the form of probabilistic Bayesian Neural Networks (pBNNs) that have the same architectures as the ANNs, except they produce interval estimates for the reconstructed intake instead of a single point estimate and improve uncertainty quantification. Epistemic uncertainty arises from a lack of knowledge or insufficient training data needed to characterize a process and may be reduced by increasing the dataset size. Aleatoric uncertainty refers to the random uncertainty due to the inherent stochasticity of the system and cannot be reduced by increased dataset size.

The following section defines and outlines how the ANNs are developed and used for each dataset tested (and described in section 2). Each individual ML model developed and tested was defined by three different regimens (r1, r2 or r3), an applied linear normalisation (8 different combinations), a model complexity (c1, c2, or c3), and model type (either ANN or pBNN). These different model configurations produced a total study design employing 24 models (8 normalisation combinations each with 3 different model complexities) per regimen. Due to computational inefficiencies and loss function instability associated with pBNNs, these model types are only developed for the optimal models identified from the 72 ANNs. Nine optimal models were distributed across the three regimens, thus resulting in a total of 81 models developed.

### 3.1. Machine learning regimens and available data features

To demonstrate the range of questions explored in this study, the following subsection outlines the various data configurations and their underlying rationale. Three regimens are presented, two of which predict the IBB and one that predicts the LTRB. Regimen 1 addresses a limited use case in which a single bioassay measurement is obtained relatively soon after the exposure with the objective of reconstructing the activity of the intake with minimal temporal bioassay data. Regimen 2 builds upon Regimen 1 but includes more simulated bioassay measurements over a longer time period generated from random sampling of activity retention equations from the ITRI annual reports; the objective of Regimen 2 was to evaluate whether data consolidation through summary statistics of activity retention equation parameters in the ITRI annual reports could yield more high-fidelity intake reconstructions from a greater number of bioassay measurements despite these additional data points being generated through a synthetic data simulation. Regimen 3 addresses the high implicit stochasticity of the Regimen 2 data and the limited number of bioassay measurements of Regimen 1 data by reconstructing the long-term retained burden based on three different cumulative absorbed dose estimates; the objective of Regimen 3 was to generate intake reconstructions with improved predictive performance than the previous two regimens by implementing the remaining available data from ITRI that are indicative of the unreported bioassay measurements. All regimens are the result of either reported or simulated data from ITRI LMF-120, of which the primary data features of interest are age in days, weight in kilograms, sex (male or female), initial body burden in microcuries, LTRB in microcuries, and time post-exposure in days. A summary of these data features are shown in table 2.

**Table 2.** Summary statistics of the input features and target outputs used in the models of the three regimens.

Parameter	Mean	Standard Deviation	%RSD	Minimum	Maximum
Age (days)	401.484	15.282	3.81%	377	438
Sex (0-Male, 1-Female)	48 M 47 F	N/A	N/A	N/A	N/A
Weight (kg)	8.741	1.232	14.09%	5.7	11.9
IBB (*µ*Ci)	592.737	740.592	124.94%	0	3000
Time Post Exposure (days)	14	0	0	14	14
LTRB (*µ*Ci kg^−1^)	27.811	33.534	120.58%	0	120

#### 3.1.1. Regimen 1: Predicting IBB from LTRB

Regimen 1 is designed to assess whether ML can reconstruct the rapidly clearing biokinetic component of an intake by leveraging available data features. As noted in section 2.1, the rapidly clearing component of the IBB relative to the LTRB exhibited high intra-group variability. The Annual Reports, particularly LF-45 [22], attribute this variability to a fraction of the inhaled strontium chloride depositing in the upper respiratory tract, where it can be rapidly absorbed into the bloodstream or cleared via mucociliary transport and swallowing, leading to radionuclide transfer to the gastrointestinal tract. Notably, the authors of the Annual Reports repeatedly caution against using IBB as a representative measure of a dog’s exposure ranking among experimental groups.

Regimen 1 consists of 95 data observations and utilises age, sex, weight, time post-exposure, and the LTRB as input features to predict the IBB target output, which examines whether ML can identify patterns or quantify uncertainty in the rapidly clearing biokinetic component of an intake. To introduce variability in the time post-exposure feature, artificial noise of ±1 day was added. Since the ITRI protocol specifies LTRB acquisition precisely 14 days post-exposure, this feature would otherwise exhibit no variance. The inclusion of artificial noise is justified by the inherent uncertainty in the timing of intake in real-world exposure scenarios. A notable example is the 1987 Goiania accident, in which caesium-137 from an abandoned radiotherapy source was inadvertently dispersed, leading to widespread contamination and delayed identification of exposure events [23]. Additionally, the ITRI experimental protocol introduces an inherent, unreported level of uncertainty in exposure timing due to batch processing of the dogs, likely on the order of hours.

#### 3.1.2. Regimen 2: Predicting LTRB from simulated data

Regimen 2 expands on the methodology of Regimen 1 by incorporating synthetic whole-body counting data to assess whether ML can reconstruct IBB under more realistic conditions with uncertainty and noise. A key challenge for constructing models as part of the study was the absence of individual whole-body counting measurements for each dog, meaning the measurements shown in figure 1 were not available for use in model development. Individual whole-body counting measurements would provide a valuable addition to an ML framework by expanding the dataset size and facilitating model development to compensate for missing data in other datasets. Although the individual measurement data was not available, Annual Report LF-39 (1967–1968) [14] tabulated the summary statistics of the non-linear regression equations developed for each individual dog when attempting to reconstruct a retention function. These retention regression equations varied in the number of exponential terms depending on the lifespan of the specific dog, where the very short-lived dogs may have had only two-component retention equations while the longer-lived dogs had four-component equations fitted to their measurement data. Each regression retention equation took the form of equation (1):

\begin{equation*} BB\left(t\right) = \sum^k_{i = 1} a_ie^{-\lambda_it}\end{equation*}

where *BB*(*t*) is a proportion of the IBB at time *t* in days, *k* is the number of exponential components to use in the retention equation, and *a_i_* and *λ*_*i*_ are the regression parameters for which the summary statistics (i.e. mean and standard deviation) are provided by the ITRI annual reports. The regression coefficients *a_i_* account for the initial body burden such that the summation of all *a_i_* values for a given dog equals the IBB (i.e. when *t* = 0). The decay constants *λ*_*i*_ account for the varying degrees of burden clearances ranging from the rapidly-clearing early time period to the gradually clearing late time period. The values for the regression parameter summary statistics are provided in table 3.

**Table 3.** Mean and standard deviation (in parentheses) of the regression parameters for individual dog retention equations.

Parameters	2 Components	3 Components	4 Components
*a*_1_	0.58 (0.03)	0.64 (0.08)	0.57 (0.09)
*λ*_1_	1.4 (0.3)	2.5 (0.8)	2 (0.5)

*a*_2_	0.42 (0.03)	0.16 (0.04)	0.15 (0.06)
*λ*_2_	0.015 (0.006)	0.031 (0.04)	0.11 (0.05)

*a*_3_		0.2 (0.04)	0.11 (0.03)
*λ*_3_		0.000 63 (0.0002)	0.0074 (0.004)

*a*_4_			0.16 (0.07)
*λ*_4_			0.000 36 (0.0002)

**Figure 3.**
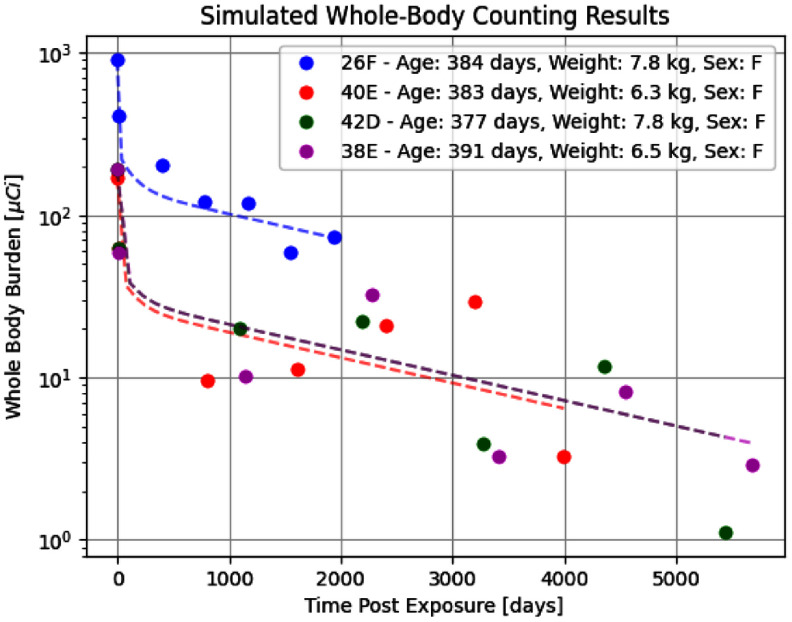
Results from the whole body counting simulation for four selected dogs with varying ages, sex, and weights. The dotted lines represent the retention regression equations for each dog without noise inclusion (i.e. no random sampling of the regression parameters).

The summary statistics for the regression retention equation parameters were subsequently updated in multiple Annual Reports with the final updates appearing in Annual Report LF-44 (1970–1971) [24]. These updates were based on recalculating the regression using data from all dogs that remained in the study at each respective reporting period. To fully leverage the information available from the summary statistics, the study implemented a simulation to generate synthetic whole-body counts for each individual dog at five evenly spaced time points between day 14 post-exposure, corresponding to the LTRB measurement, and the recorded death date for each dog. The simulation modelled each *a_i_* and *λ*_*i*_ as independent Gaussian distributions, with synthetic measurements generated by randomly sampling from these distributions, which represent retention parameters for each dog. To maximise applicability across the dataset, the simulation utilised summary statistics from Annual Report LF-39 (1967–1968) [14], since regression parameters from subsequent reports would not be valid for dogs that had already died by the corresponding reporting periods. This simulation method artificially increased the dataset from 95 reported measurements of LTRB to 288 measurements. Dataset augmentation through the use of limited-knowledge simulations is employed in the fields of bioinformatics [25] and computer vision robotics [26], which report improved performances in cases where the number of real observations is small compared to the number of data features and when the simulation data is paired with the limited real data; the current study fulfils the first criterion and pairs the simulated whole body counting measurements with the LTRB (i.e., the real data). The simulation was only conducted on the 48 longevity dogs that were not part of the control group, which gives rise to the 288 observations, 48 of which are true measurements offered by the LTRB in the annual reports, and the remaining 240 are the five evenly spaced synthetic whole-body counting measurements generated for the 48 dogs. Figure 3 contains results of the data simulation for four dogs, whose age, sex, and weight are noted in the legend; the dotted lines are the retention equation for each of the four dogs absent of any statistical noise, while the corresponding data points show the result of randomly sampling all regression parameters needed for each of the four dogs’ retention equation. For the dog denoted by the green marker, 42D, the retention equation is the same as that for dog 38E due to similar lifespans and IBBs, which results in the purple dotted line covering the green. Note that the four dogs shown are the same as those selected by the investigators at ITRI in figure 1. Comparison of the simulation data points of figure 3 with those in figure 1 reveals two qualitative observations. The first is the notably higher variability in retention within individual dogs in the simulated data compared to the true data from the figure originating from the historical data. This outcome naturally arises from using summarised regression parameters, which incorporate variability across individual dogs, to model retention for each case. The second observation is the lower variability between individual dogs in the noiseless retention equations of figure 3 relative to the historical data; this observation is a consequence of the summarised parameters effectively constraining the categorisation of dogs into one of three retention equations based on lifespan.

In summary, Regimen 2 utilises the combined reported and simulated dataset to evaluate whether ML can reconstruct the IBB, similar to Regimen 1, using whole-body counting data with a realistic degree of uncertainty and noise, primarily driven by aleatoric variability.

#### 3.1.3. Regimen 3: Predicting LTRB from historical absorbed dose estimates

Regimen 3 extends the analysis by incorporating dose estimates from the Annual Reports to evaluate whether ML can reconstruct the retained activity based on reported biokinetic data. The final regimen of the study sought to leverage the dose estimates included in the Annual Reports, as they were one of few data that were reported at different time points. ITRI Annual Report LF-39 details the dosimetric estimation method as an absorbed beta dose to the skeleton. This absorbed dose was calculated according to equation (2):

\begin{equation*} \text{Absorbed } \beta \text{dose (rad)} = \frac{0.457A_0}{W}\int^t_0 \mathrm{SB}\left(t\right) \mathrm{d}t\end{equation*}

where *A*_0_ is the initial body burden (IBB) of $ ^{90}\mathrm{Sr}$ in *µ*Ci, *W* is the total body weight of the dog when it reached adulthood, and $\mathrm{SB}(t)$ is the average skeletal burden at time *t* expressed as a fraction of the initial body burden. Equation (2) was formulated under the assumptions that the skeletal weight is equal to 10% of the total body weight, the total body weight remained constant throughout the observation period, and the average skeletal burden could be adequately expressed as a the whole-body retention minus the first (i.e. early) clearance component [14].

### 3.2. Neural network frameworks

Two different types of neural networks were applied as complements to one another. Of the 81 models developed, 72 belonged to the ANN framework and 9 to the pBNN framework. The ANNs function as regression neural networks composed of fully-connected layers of nodes that eventually collapse into a single terminal output node, depending on the regimen and desired target output. These networks produce point estimates for the target outputs, but provide little information on the degree of uncertainty associated with each individual prediction.

Due to this limitation of traditional ANNs, the study adopted pBNNs, which are an advanced subset of neural networks well-suited to learning from data that arises from an inherently stochastic process. The pBNNs introduce two fundamental differences from ANNs. The first modification is that rather than modelling the synapses between nodes of adjacent layers as scalar values, a Bayesian neural network (BNN) models the synapses as probability distributions that must be learned during the training. Consequently, any request for a new prediction from the network during the testing phase involves randomly sampling at every synapse. This approach incorporates epistemic uncertainty, which arises from a limited or insufficient training dataset. The second modification is to the formulation of the desired target output. Rather than progressively reducing the number of nodes in subsequent layers until reaching a single output node, the final layer of the pBNN consists of two nodes: one for the mean of the output prediction, and the other for the standard deviation.

To produce an output prediction that represents repeated sampling from the distributions of synapses for pBNNs, the loss function is changed from the mean squared error (MSE), which is used in ANNs and shown in equation (3), to the negative log-likelihood ($\log\mathbb{P}$) function shown in equation (4). Thus, the synaptic weight distributions are optimised to maximise the probability that the true output value of intake is drawn from the distribution defined by the mean and standard deviation predicted by the network [27]

\begin{equation*} \text{MSE} = \frac{1}{n}\sum^n_{i = 1}\left(y_i-\hat{y}_i\right)^2\end{equation*}

\begin{equation*} \text{log}\mathbb{P}\left(\mathcal{D}|\theta\right) = \sum^n_{i = 1} \left(y_i\text{log}\left(\hat{y}_{\theta,i}\right) + \left(1-y_i\right)\text{log}\left(1-\hat{y}_{\theta,i}\right)\right)\end{equation*}

where $\hat{y}_i$ is the predicted output and *y_i_* is the true output in equation (3). In equation (4), $\mathbb{P}(\mathcal{D}|\theta)$ is the probability of the observed dataset $\mathcal{D}$ being predicted given the neural network with the set of trainable parameters *θ*, *n* is the number of training observations in the dataset, *y_i_* is the true output for the *i-*th training observation, $\hat{y}_{\theta,i}$ is the predicted output for the *i-*th training observation when computed using the neural network with the set of trainable parameters *θ*. In this context, *θ* denotes a set of trainable parameters, which include synaptic weights and biases. For pBNNs, *θ* represents a set of synaptic distributions sampled to generate output predictions. Since the pBNNs rely on synaptic distributions, prior and posterior distributions had to be initialized. The prior distribution over synaptic weights was defined as a multivariate Gaussian with mean 0 and unit variance, reflecting an uninformed assumption about their form. The posterior distribution was not assumed directly but was instead approximated during training using variational inference, which iteratively adjusts a parameterized distribution to minimize the Kullback-Leibler (KL) divergence from the unnormalized posterior.

### 3.3. Linear normalisation groups

Although each regimen used different inputs and outputs, all regimens implemented five input features to predict a single output. Linear normalization, or min-max scaling, is a preprocessing technique that reduces the risk of high-magnitude features dominating in predictive contribution to the trained model. The imbalance of feature contributions is remedied by scaling to a bounded range of 0 to 1. Such techniques are especially beneficial for neural networks and have been shown to improve performance for regression tasks [28]. For the implemented dataset, the use of normalization was justified because the IBB and age carried much higher value ranges than other features. To systematically determine the optimal application of linear normalisation during preprocessing—without exhaustively testing every possible combination for each variable—the input and output parameters were grouped into three categories. Group A comprised features related to the physiology and anatomy of the exposed individual: age, sex, and weight. Group B consisted of the single chronological variable, time post-exposure. Group C contained all features associated with internal radioactive exposure in the Beagles, which, depending on the regimen, incorporated the IBB, LTRB, simulated whole-body counts (Regimen 2), and estimated skeletal absorbed dose (Regimen 3). These three normalisation groups defined eight possible preprocessing configurations, ranging from no normalisation to normalisation of all variables. The typical value ranges of the inputs and outputs used are shown in their native units in table 2.

### 3.4. Network hyperparameters

The study adopted three different model architectures of increasing complexity. The low, moderate, and high model complexity architectures were defined as [5–10–3–1], [5–10–9–7–5–3–1], and [5–10–15–14–13–12–10–9–8–7–6–5–4–3–1] with 97, 548, and 1443 learnable parameters or distributions, respectively. Each of the numbers in the bracketed notation represents the number of nodes in the fully connected layers, meaning the low complexity has an input layer with five input nodes, a hidden layer with ten nodes, another hidden layer with three nodes, and an output layer with a single node. Despite the low dimensionality of the input matrix, the high complexity architecture was included as an upper bound to explore a wide range of model complexities, given the lack of prior literature on intake reconstruction using neural networks. To mitigate potential overfitting arising from pairing high complexity with low-dimensional inputs, L2 regularization was implemented and compared against non-regularized models. Since neither the predictions nor the performance metrics exhibited noticeable differences, the results discussed correspond to the non-regularized models. The activation functions used at the nodes were either the rectified linear unit function (ReLU) or the SoftPlus function. The number of epochs, or training iterations, was varied to achieve a convergence criterion of less than 0.5% relative change in the training loss. For the ANNs, the lowest and highest relative change in the training loss across all three regimens was 0.0002% and 0.49%, respectively. The number of epochs spanned from 100 to 1000, where the higher number of epochs was typically used for the high-complexity model architectures paired with the pBNN network type. The optimiser function used for all models was the adaptive moment estimation function (ADAM) with an initial learning rate that varied from 10^−5^ to 10^−1^ and was tested by changing the order of magnitude [29]. Generally, the models with a normalised output necessitated the lower initial learning rates. The training/testing split varied between using 85% or 90% of the dataset for training. The combination of hyperparameters that produced the results in the next section was recorded for every individual model.

### 3.5. Model performance criteria

The merit of each model was evaluated according to a two-parameter optimisation meant to simultaneously maximise the accuracy and variance consistency. The primary evaluation metrics were:

1. **Root Mean Square Error (RMSE)**—Measures prediction accuracy, where lower values indicate better performance.
2. **Variance Relative Difference (VarRD)**—Assesses the consistency between the variance of the model predictions and the true variance observed in the experimental data. Lower values indicate lower model bias.

Each of the three regimens has two plots that summarise the results. The right side of each figure depicting the results for each regimen includes the two-parameter optimisation visualised where:

• The ***x*-axis** represents the inverse of RMSE, where higher values indicate better accuracy.
• The ***y*-axis** represents the inverse of VarRD, where higher values indicate lower model bias.

Expanding upon the second metric, the VarRD is formally defined as the difference between the variances of the predicted and true values relative to the variance of the true values as shown in equation (5), where *σ*^2^ is the variance of the test set true outputs or the predictions made from the test set inputs. A negative VarRD signifies that the variance of the model predictions are less than that of the true values, which may suggest a biased model depending on the magnitude of the difference. The use of the term bias in this context differs slightly from statistical bias, which is defined as the difference between an estimator obtained from a sample and the true population parameter. In machine learning, however, bias typically refers not to the model’s learned parameters but to the predictions themselves. A model that produces predictions with low variance if often biased toward outputting similar values, whereas predictions with high variance may indicate overfitting. This dichotomy exhibits the bias-variance tradeoff [30]. Because empirical bias is difficult to quantify directly, the variance relative difference (VarRD) metric serves as a practical surrogate to indicate where a given model lies along the bias-variance spectrum [31]. The inclusion of the VarRD and taking the inverse amplifies the subtle differences in model biases such that the most biased models are omitted from the Pareto front of the two-parameter optimisation. The Pareto front, a concept from optimisation, refers to the set of solutions for which no other solution is strictly better in all considered metrics. In other words, a solution is on the Pareto front if it cannot be improved in one objective without sacrificing performance in another [32]. For this study, the optimal solutions refer to models that are not simultaneously exceeded in inverse RMSE or inverse VarRD by any other models.

Generally, the two-parameter optimisation plots may be interpreted as accuracy improving towards the right on the *x*-axis and model bias decreasing moving upward on the *y*-axis. The analysis taking the form of this two-metric optimisation exemplifies consideration of the model performance along the bias-variance trade-off [30]. For each of the three regimens, the optimal models are selected from the Pareto front of each two parameter optimisation and analysed using the following visualisations:

1. **Scatter Plot (Training Set):** Displays predicted values against true values with a perfect agreement diagonal and a regression line to visually assess model fit.
2. **Residual Plot (Test Set):** Compares model predictions to true values, with a 25% relative error margin to evaluate generalisation performance.
3. **Loss Convergence Plot:** Illustrates RMSE evolution over training epochs for both training and testing sets, ensuring model convergence.

Due to the high sensitivity and implementation complexity of pBNNs, the two-metric optimisation was not performed for these architectures. Instead, each pBNN was trained using the complexity and normalization settings of its corresponding optimal ANN. Hyperparameter tuning to improve performance was still conducted independently for each pBNN.

\begin{equation*} \mathrm{VarRD} = \frac{\sigma^2_\mathrm{predicted} - \sigma^2_\mathrm{true}}{\sigma^2_\mathrm{true}}.\end{equation*}

## 4. Results

### 4.1. Regimen 1: reconstruction the initial intake (IBB) through whole-body counting measurement soon after exposure (LTRB)

**Figure 4.**
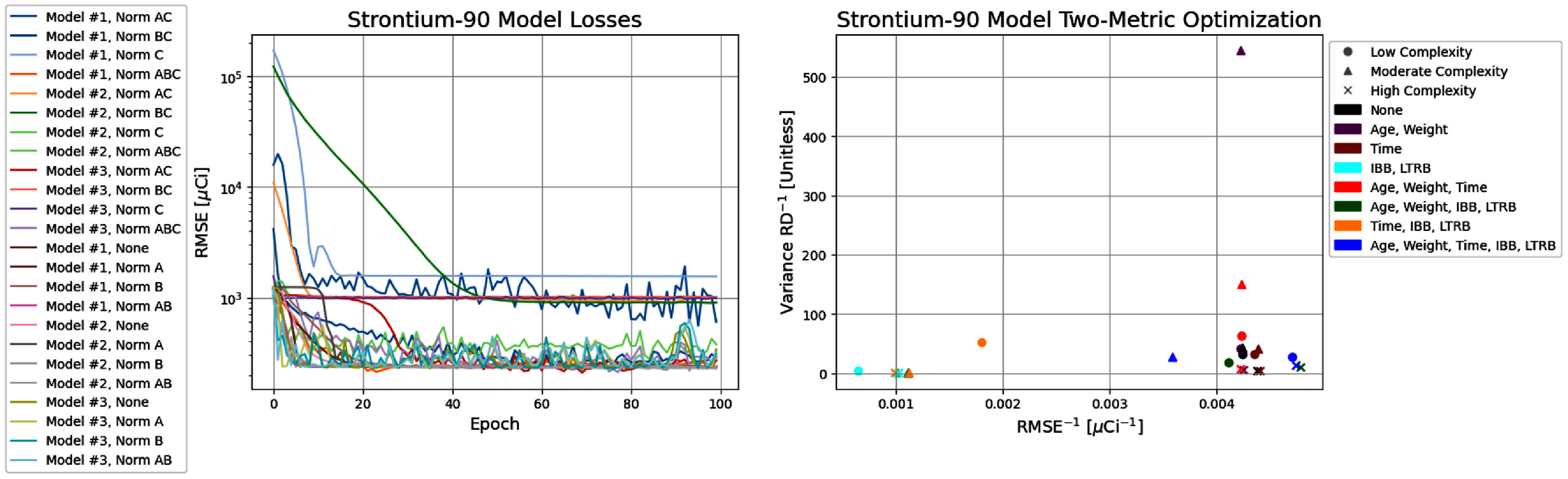
Progression of loss functions (left) for all ANN models of Regimen 1 and the accompanying two-parameter optimisation (right).

**Figure 5.**
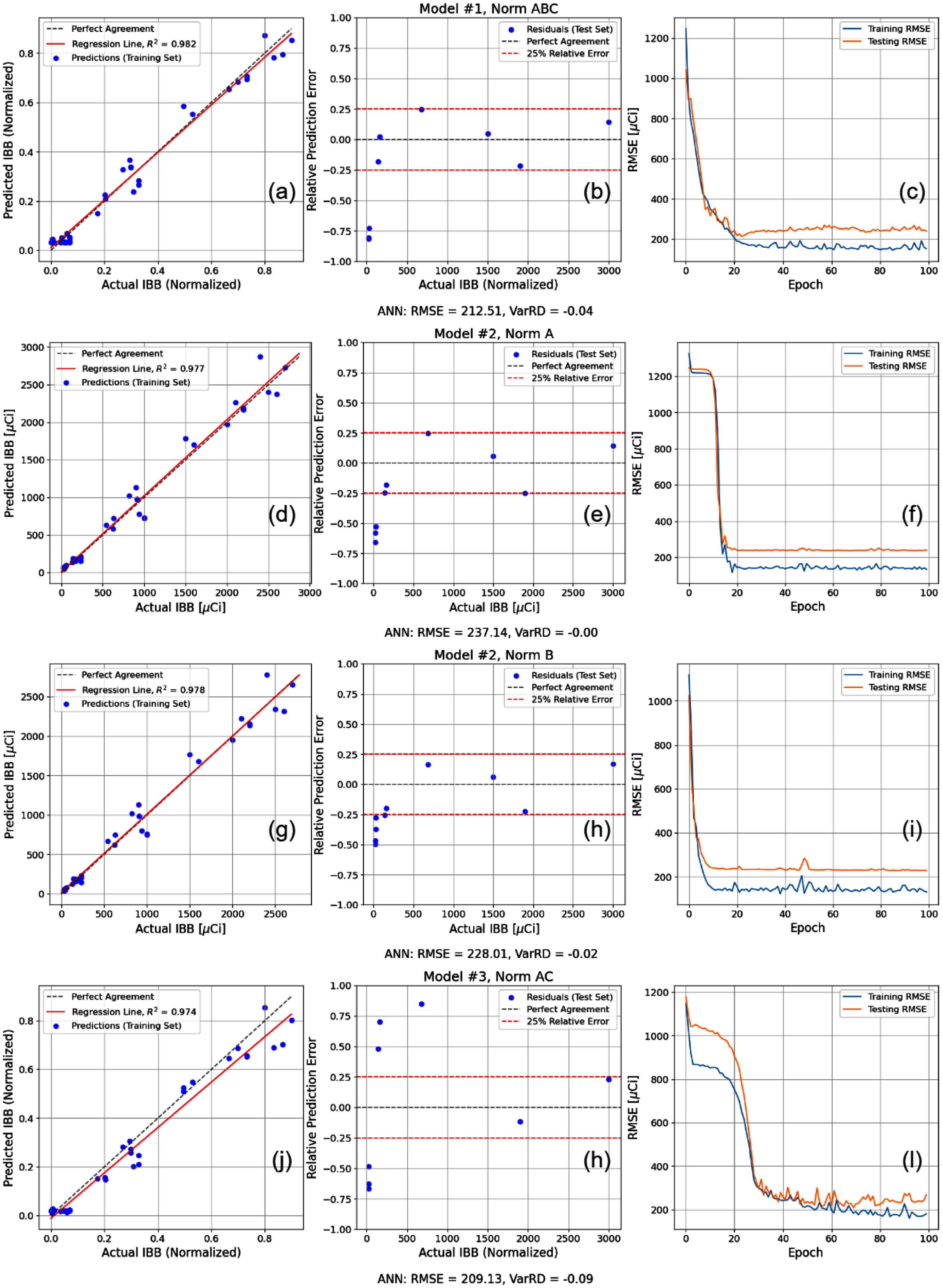
Optimal models of Pareto front in optimisation of figure 4. Models labelled by complexity and normalisation (i.e. Model #1, Norm ABC) and correspond to row of plots directly below label. (a), (d), (g), (j) Predicted IBB as function of actual IBB for the training set. (b), (e), (h), (k) Relative error of IBB predictions as function of actual IBB for test set. (c), (f), (i), (l) Training and test losses as function of epoch.

Regimen 1 sought to reconstruct the intake activity by inputting the age, sex, weight, time post-exposure with artificial noise of ±1 day to determine whether a single bioassay measurement post-exposure is sufficient for the activity reconstruction. Figure 4 shows both the model losses and the two- metric optimisation for all models developed in Regimen 1. The most important data points of the two-metric optimisation are those that are not simultaneously exceeded in both the horizontal and vertical axes, meaning they lie along the Pareto front; thus, the clustering of points of non-optimal solutions are not discussed. The two-parameter optimisation (figure 4) shows little dependence of the overall model performance on the normalisation applied, with the exception of an absent normalisation (None) being a sub-par preprocessing technique, meaning the data does require some degree of normalisation due to the high difference in orders of magnitude of the features, which is consistent with ML implementation in other scientific domains, and more specifically, with ANNs. In addition, the lower model complexity tends to be inadequate for capturing a pattern in the rapidly-cleared biokinetic component of the initial inhalation exposure. The moderate complexity excels at eliminating bias in the predictions, suggesting that the high complexity is unnecessary for ensuring consistent variance. The individual results shown in figure 5 show that the lowest model complexity with complete normalisation (Model 1, Norm ABC) results yields the most balanced performance with regard to accuracy (RMSE of 212.51 *µ*Ci) and variance (RD of −4%), and the model is capable of learning the data with a regression that lies nearly atop the perfect agreement line.

Although the highest model complexity (Model 3, Norm AC) has a marginally lower RMSE of 209.13 *µ*Ci, it suffers from bias at lower values, and tends to underestimate intake activities while simultaneously requiring more training iterations to converge. As suggested by the two-metric optimisation and confirmed by the individual results, the moderate complexity exhibits the most prompt convergence, consistent variance, and high accuracy while applying the lowest amount of preprocessing. Each of the optimal models implementing the moderate complexity have RMSEs of 237.14 and 228.01 *µ*Ci and variance RDs of 0% and −2% when normalising either age, sex, and weight (Model 2, Norm A) or normalising time post-exposure (Model 2, Norm B). The latter exhibits a regression line that coincides best with the perfect agreement line compared to the other optimal models and has the highest fidelity predictions on the test set; the only predictions exceeding a 25% relative error are at the low intake activities which is a flaw shared among all models. However, the use of a relative difference for depicting the accuracy of the test predictions inherently penalises predictions at lower initial body burdens more than the high intake counterparts, even though accuracy at the higher intake activities is likely more consequential in an exposure scenario.

### 4.2. Regimen 2: reconstruction of initial intake (IBB) through simulated whole-body counting measurement and LTRB

**Figure 6.**
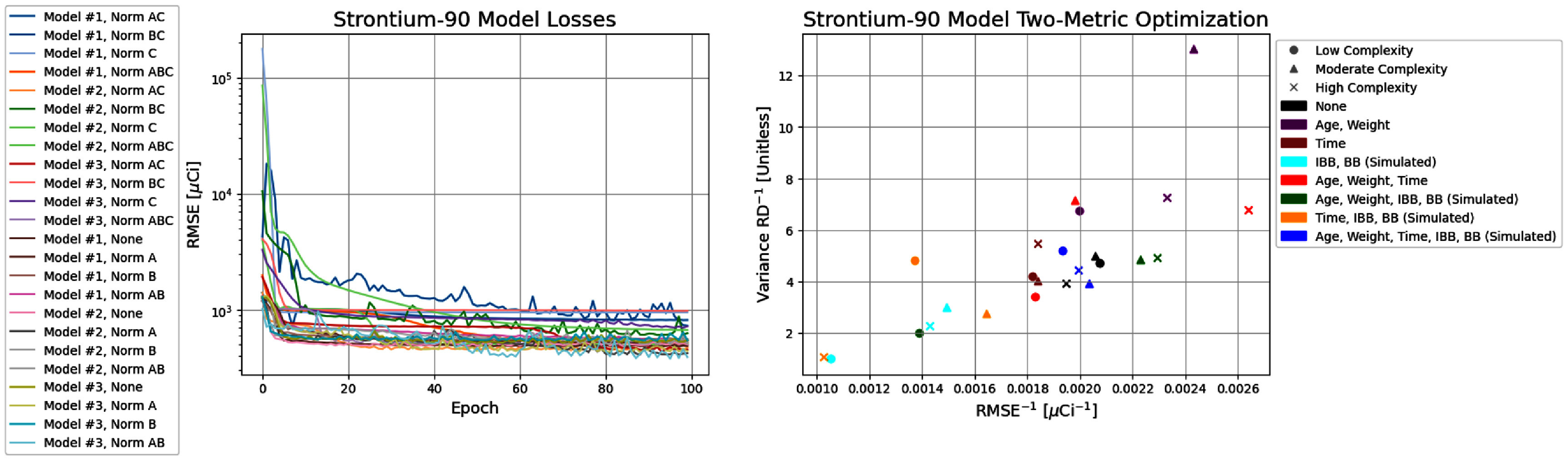
Progression of loss functions (left) for all ANN models of Regimen 2 and the accompanying two-parameter optimisation (right).

**Figure 7.**
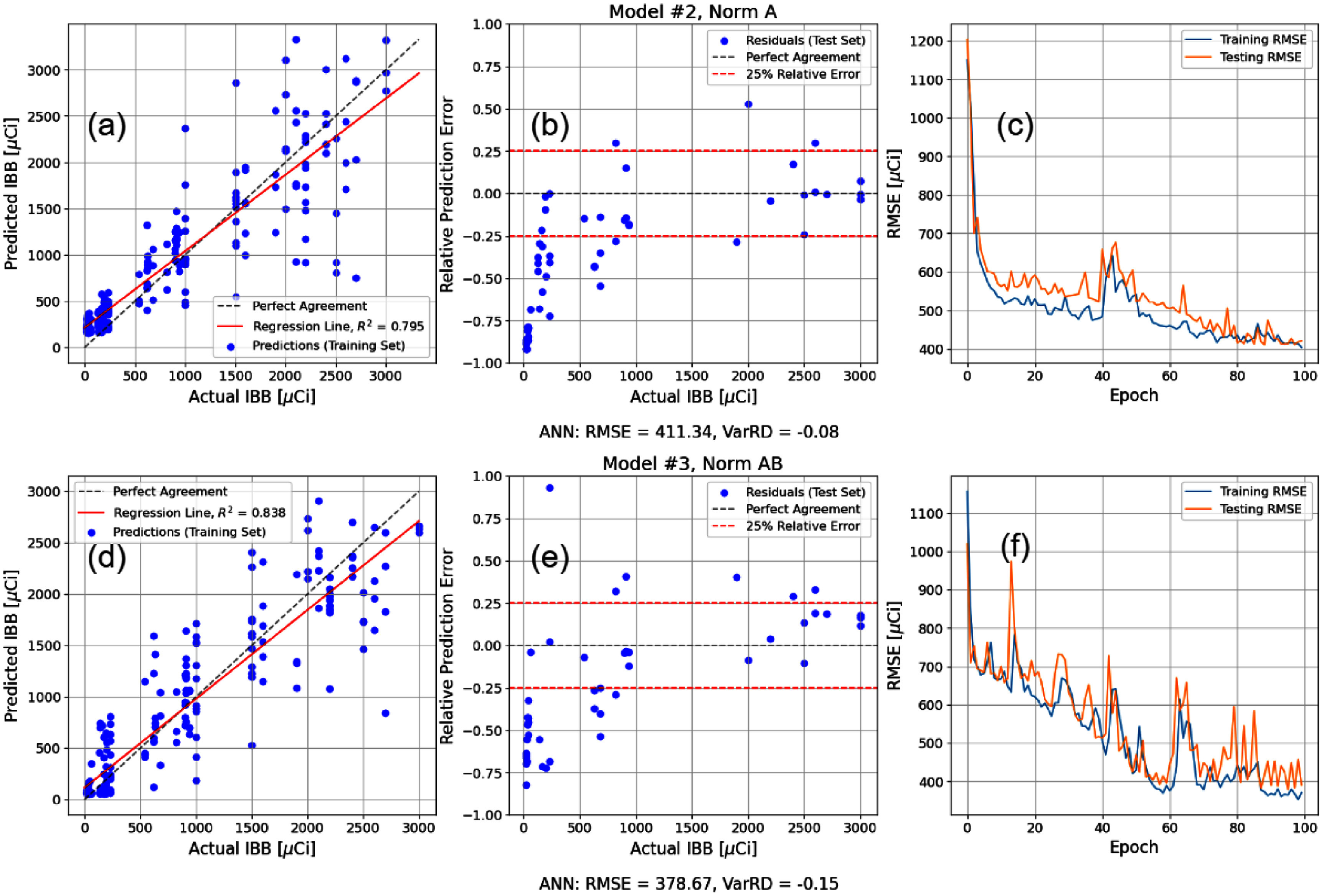
Optimal models of Pareto front in optimisation of figure 6. Models labelled by complexity and normalisation (i.e. Model #2, Norm (A) and correspond to row of plots directly below label. (a), (d) Predicted IBB as function of actual IBB for the training set. (b), (e) Relative error of IBB predictions as function of actual IBB for test set. (c), (f) Training and test losses as function of epoch.

Regimen 2 used the combination of the LTRB and the synthetic whole-body counting measurements from the statistical data simulation along with the physiological inputs to reconstruct the intake activity with the objective of both increasing the training set size and determining whether the summary statistics of the retention equation parameters could yield improved predictive performance. Figure 6 shows the two-metric optimisation for Regimen 2 where accuracy is maximised on the horizontal axis and bias is minimised by the vertical axis. The moderate and high complexity models emerge as the highest performing, especially with regard to reconciling the variance of the predictions to match that of the actual values, while the low complexity models do not exhibit an optimal performance for either metric. This is consistent with the prior information of the relatively large statistical noise associated with the simulated whole body counts, which cannot be adequately captured by the two hidden layers of the low complexity models despite the preprocessing implemented. Unlike the several models of Regimen 1, two models define the Pareto front for Regimen 2. The first is a moderate complexity model that normalises age and weight, which excels in the consistency of the variance of predictions with the true set. The second is the high model complexity that normalises age, weight, and time post exposure. It is also noteworthy that the third most optimal model (also high complexity) also happens to normalise age and weight, further exemplifying the utility of this normalisation choice on the performance of models. The models that do not normalise age and weight consistently perform the worst on Regimen 2, with the exception of the low complexity that normalises age, weight, IBB and simulated whole body burden. The individual performances of the two optimal models are shown in more detail in figure 7. The model with the most consistent variance of predictions (Model 2, Norm A) exhibits a VarRD of −8% and an RMSE of 411.34 *µ*Ci. The most accurate model (Model 3, Norm AB) has an RMSE of 378.67 *µ*Ci and a VarRD of −15%. Similar to the optimal models of Regimen 1, both highlighted models of Regimen 2 suffer from an underestimation of IBB at the lower values of intake activity, mostly existing in the range of 0–500 *µ*Ci and to a lesser degree from 500–1000 *µ*Ci. However, in contrast to Regimen 1, this trend is seen in the test set but not the training set, meaning the regression line showing how well the models learn the data would indicate an overestimation at the lower values of intake activity. Regardless, the left-hand plots of figure 7 show that the simulated whole body counts (simulation results shown in figure 3) generated from the summarised regression parameters of table 3 have an excessive degree of noise. In particular, for many of the longer-lived dogs, the simulation must randomly sample from eight parameters (four coefficients and four decay constants) for each prediction made for a singular dog. This excessive random sampling leads to many predictions made as part of the same time series coming from different parameters. Therefore, a more accurate simulation would require individualised regression parameters from the whole body counts from each dog, however, these data are absent from the ITRI Annual Reports and were unable to be found in the literature. Superior to individualised regression parameters, however, would be individual records of the whole-body counting data.

### 4.3. Regimen 3: reconstruction of retained activity through skeletal absorbed dose estimates for three post-exposure time points

**Figure 8.**
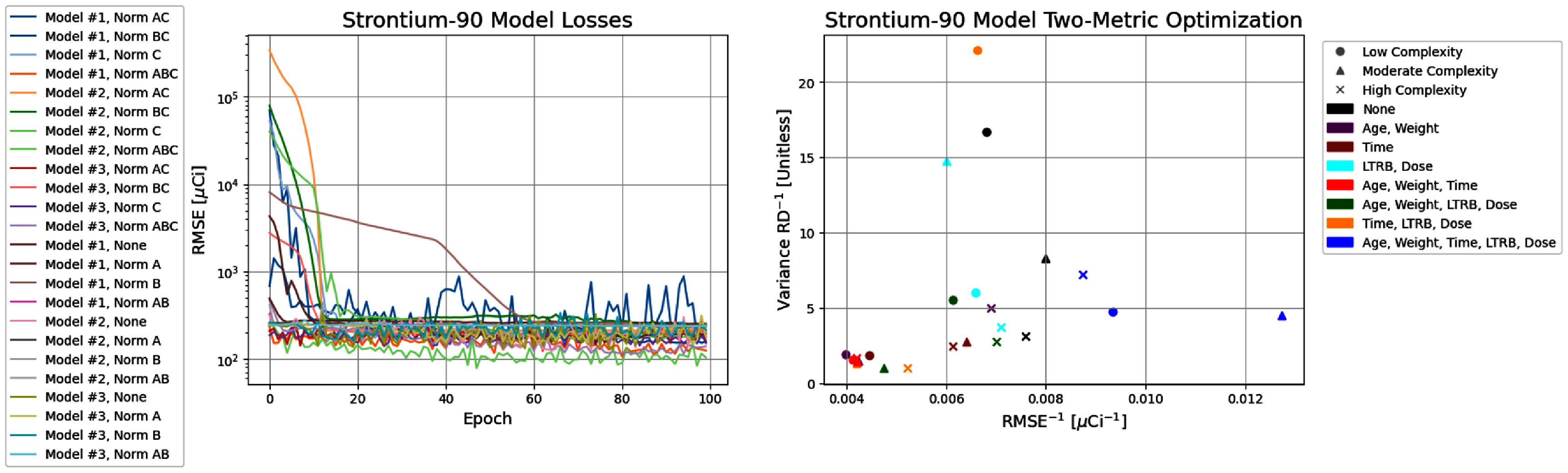
Progression of loss functions (left) for all ANN models of Regimen 3 and the accompanying two-parameter optimisation (right).

**Figure 9.**
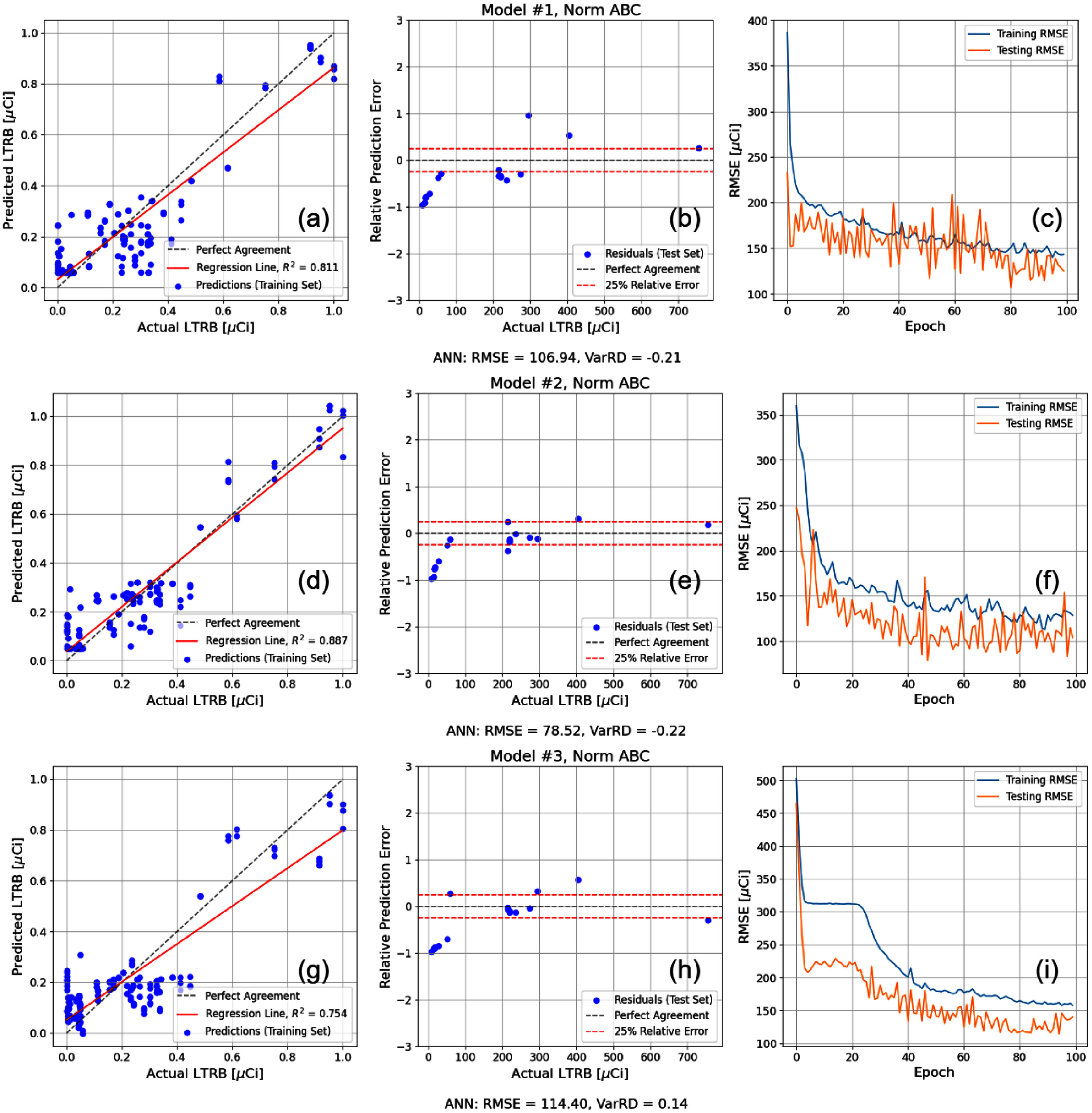
Optimal models as defined by Pareto front in two-metric optimisation of figure 8. Models defined and labelled by complexity and normalisation (i.e. Model #1, Norm ABC) and correspond to row plots directly below label. (a), (d), (g) Predicted IBB as function of actual IBB for the training set. (b), (e), (h) Relative error of IBB predictions as function of actual IBB for test set. (c), (f), (i) Training and test losses as function of epoch.

Regimen 3 models were developed to reconstruct the retained activity (i.e. the LTRB) based on the three absorbed dose estimates and the physiological inputs provided by the ITRI annual reports with the objective of producing accurate predictions without the reliance on simulated data as in Regimen 2 while increasing the training set size relative to Regimen 1. Regimen 3 offers the highest amount of optimal models as defined by the Pareto front in the two-parameter optimisation of figure 8, which shows the consolidated results of the models developed as part of Regimen 3. Only 3 of the 6 optimal models are shown in figure 9; this is due to extraneous regression lines of actual versus predicted values revealing low predictive ability of the model despite having relatively competitive accuracy and consistency of predicted variance. The three models deemed adequate within the larger set of optimal models were chosen without consideration of model architecture or normalisation. The lowest model complexity is the most well-represented along the Pareto front with three configurations: one with no normalisation, one with normalised time post exposure, LTRB and absorbed dose and, lastly, one with complete normalisation. The three models excluded from the individual results shown had lower relative differences between the predicted and actual variances, but this typically came at the expense of the model accuracy. As such, the three selected models, all of which implemented complete normalisation, performed moderately well at replicating a realistic degree of variance in the calculated absorbed dose and had high accuracy. From the results of the three selected optimal models, it is apparent that despite the high complexity and low complexity models having slightly better agreement with the actual variance of the dataset, with VarRDs of 14% and −21%, respectively, the moderate complexity is overall the best model. The moderate-complexity model exhibits a variance RD of −22%, which is on par with those of the aforementioned models, while having a significantly better accuracy as measured through the RMSE of 78.52 *µ*Ci. In addition, the regression of predicted versus actual values has the highest R-squared of 0.887, showing the moderate complexity best learns the data. Its plot of test predictions contains the highest number of predictions within the 25% relative error margin further proving its superior predictive capability over the other two models. Of note, the highest model complexity has the best variance RD, which is likely due to the model overfitting to the data points which leads to diminished performance on the test set while its regression line still suggests that the model would tend to underestimate dose for the higher magnitude values of calculated absorbed dose.

### 4.4. Probabilistic neural network versions of optimal ANN models

**Figure 10.**
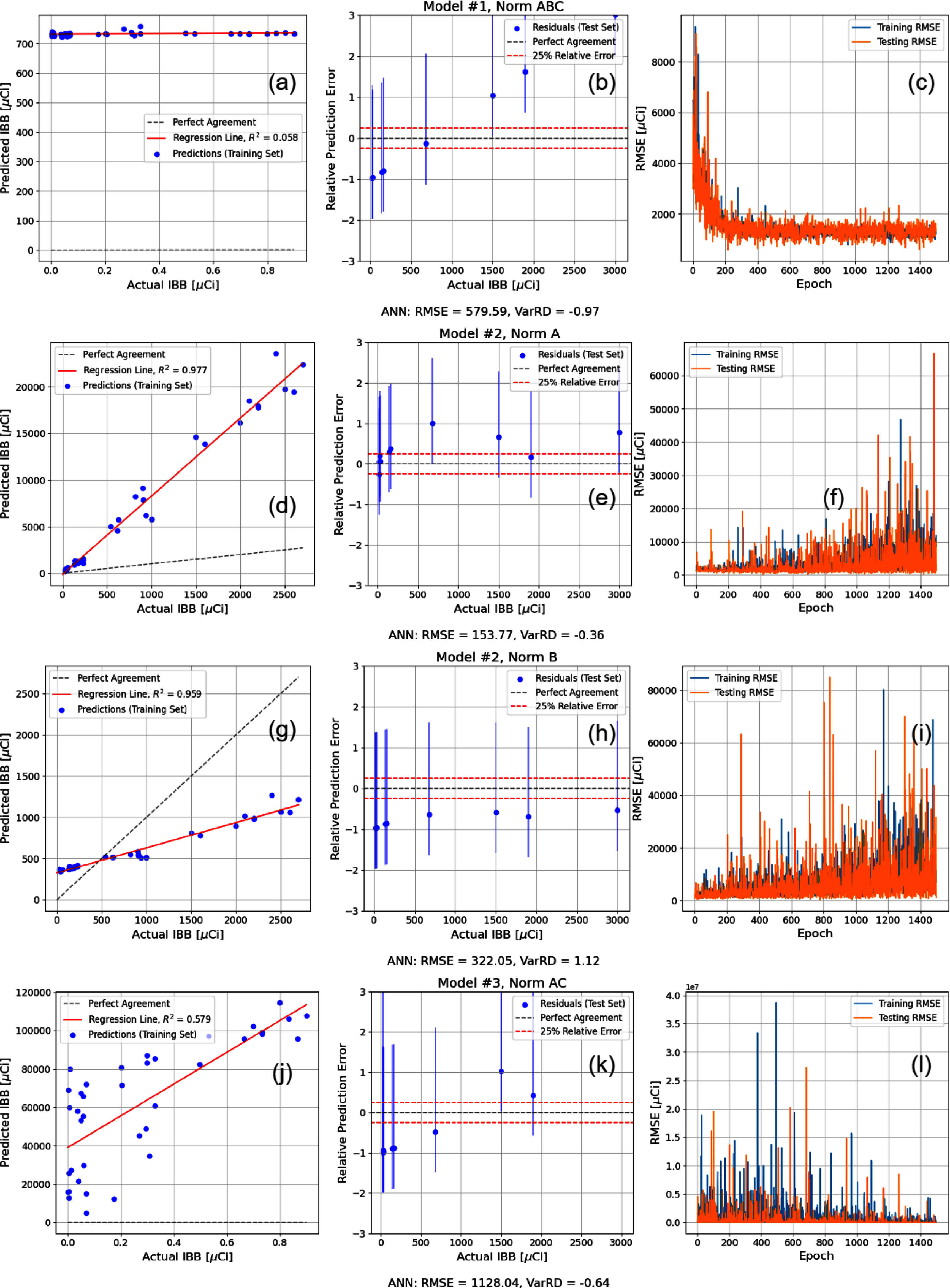
The pBNN analogues to optimal models of Regimen 1 in figure 5. Models defined and labelled by complexity and normalisation (i.e. Model #1, Norm ABC) and correspond to row plots directly below label. (a), (d), (g), (j) Predicted IBB as function of actual IBB for the training set. (b), (e), (h), (k) Relative error of IBB predictions as function of actual IBB for test set. (c), (f), (i), (l) Training and test losses as function of epoch.

The pBNNs in this study were trained only for the optimal model configurations determined from the 72 ANNs. This selection was made due to the individual difficulties of training pBNNs including the extremely high epistemic uncertainty from such limited training data, which inherently makes learning the synaptic distributions either computationally inefficient or prone to convergence upon very broad distributions, which results in large confidence intervals for the outputs of the test set. Additionally, the negative log likelihood function is a sensitive loss function in which normalisation can heavily impact its ability to converge on adequate parameters. Figure 10 shows the individual model results for the four optimal models of Regimen 1. The training and testing losses for both iterations of the moderate complexity model (Model 2), each of which normalises only one group of variables, do notconverge consistently. This reinforces the conclusion that widespread normalisation may be needed in cases where the volume of data is low, as the low complexity model has the most uniform convergence of testing and training loss, likely due to the complete normalisation of the data. Despite this convergence, the low complexity model still produces biased predictions, as the low depth of the neural network does not offer enough learnable parameters, or synaptic distributions, to encompass the highly uncertain system. The high complexity model’s loss plot suggests a slight convergence and does not appear to over- or under-fit based on the general alignment of the training and testing losses. The high complexity model exhibits the highest variance of the predictions, as expected, but the accompanying confidence intervals for the test set reveal the concurrent low accuracy (also shown by the 1128.04 *µ*Ci RMSE).

## 5. Discussion

This study proposed a generalised framework for investigating ML capabilities for processing historical data from internal dosimetry studies by implementing multiple network architectures, data pre-processing techniques, and advanced extensions of regression-focused neural networks to represent the high degree of inherent uncertainty.

Using this framework, Regimen 1 results demonstrated that despite the cautionary text of ITRI Annual Report LF-38, which states that ‘groupings by initial body burden may be meaningless by 14–30 days post-exposure’ [33], ML may be capable of reconstructing the initial intake activity, especially in the early period post-exposure, assuming the detection methods employed at ITRI were reliable. The results demonstrated that the initial intake activity may be reconstructed using ML with strong quantitative agreement when subsequent whole-body counting measurements are made soon after the exposure, within 14±1 days.

Regimen 2 served as a critique of the summary statistics of table 3 that were offered in place of the individual dog-specific retention regression equation parameters. Since these summarised parameters drove a realistic simulation to recover some measure of detected activity through an individual dog’s lifetime, the results of the simulation represented the noisiest and highest-variance activity measurements that would be realised in a new internal exposure scenario. A notable limitation of the statistical data simulation is the assumption of a temporally uniform uncertainty in the body burden. Previous literature notes that uncertainty in body burden generally decreases over time, particularly when increased sampling frequency is employed after the discovery of an exposure [34, 35]. Implementing a temporally decreasing uncertainty term in the simulation would likely increase realism and robustness of the generated data. Nonetheless, the simulation was unified with the statistical prowess of neural networks to investigate if the deep learning framework could reconstruct the IBB given multiple activity measurements throughout the life of the dog rather than a single measurement as in Regimen 1. From this approach, the summarised regression parameters lacked the resolution to reliably train a deep learning network. This accentuates the importance of retaining individualised data for both epidemiological research and ML development. A recommendation from this finding would have future animal studies record individualised activity measurements and individual retention regression equation parameters, if such a regression is performed at all.

Lastly, Regimen 3 sought to explore the capability of ML to replicate the historical methods of dose estimation, which can be used for augmenting the literature with dose estimates for dogs that died early or were part of the sacrifice study, as opposed to the longevity study. From this approach, it was concluded that complete normalisation was needed to accomplish the task, and the best results were achieved through the moderate-complexity model. However, the greater number of low dose estimates from the dogs with low IBB living longer biased the predictions to be underestimated at higher magnitudes of estimated absorbed dose. Furthermore, updating the literature with dose estimates according to contemporary methods would serve well to create a direct link between activity measurements post-exposure and dose estimates, which, if extrapolated eventually to humans, could prove an invaluable tool in internal exposure cases where prompt estimation of dose is necessary for appropriate response and action.

The pBNNs best reveal the challenges facing data needs and quality within the field of internal dosimetry and biokinetic modeling. For ML to contribute to characterising the uncertainty that is observed through measurements, experimental design and reported data must account for the high volume of data needed for ML. Specifically, many individual observations, increased input features (such as organ specific activity measurements), and highly individualised data (in contrast to summary statistics for experimental groups, which are the typical values reported for radiobiological studies) are needed. While these data needs inevitably challenge experimental design, the resulting measurements and features would enable an ML to expedite investigations of new biokinetic systems or make individual-level predictions for personalised intake reconstruction or to better inform radiological emergency response. Overall,this study recommends that the most viable model for reconstructing acute intake activity following an inhalation is a moderate-complexity ANN requiring the inputs of age, sex, weight, approximate time post-exposure according to the exposed individual and a whole body counting measurement taken within 14 days of the exposure.

## 6. Conclusions

This study introduces the historical ITRI beagle dataset derived from controlled inhalation exposures to soluble ^90^Sr, with the accompanying experimental procedures and available datasets, to evaluate the feasibility of ML-based reconstruction of internal dosimetry metrics using both reported and synthetically augmented datasets across three defined regimens. Following the review of this historical documentation, the statistical data simulation to generate synthetic whole-body counting measurements was justified as a means of data augmentation and exploratory reconstruction. These data, whether reported or simulated, underpinned the development of a systematic ML framework composed of three regimens defined by distinct input-output configurations, but with similar approaches to normalisation and model complexity scaling.

Regimen 1 employed only the reported data to reconstruct the intake activity (i.e. IBB) with the physiological inputs and bioassay measurement of activity taken approximately 14 days post-exposure (i.e. LTRB). This configuration demonstrated that ML can replicate traditional intake reconstruction with strong quantitative agreement, achieving a best model RMSE of  212.51 *µ*Ci. Regimen 2 addressed the low training data of Regimen 1 by implementing a hybridised input combining the real data (i.e. LTRB) with the synthetic whole-body counting measurements from the aforementioned statistical data simulation. Despite the expanded dataset, the summarised activity-retention regression parameters available in the ITRI annual reports proved insufficient to yield high predictive performance of intake reconstructions, underscoring that the synthetic data could not preserve intra-subject kinetic fidelity. This finding reinforces the necessity for radiobiological and biokinetic studies to maintain individual-level temporal data for valid machine learning implementation. Regimen 3 simultaneously addressed data needs in the prior two regimens by utilising the absorbed dose estimates from the ITRI annual reports to reconstruct the activity retained after 14 days post-exposure. The Regimen 3 models achieved high predictive performance, but were biased towards lower dose group cohorts due to their longer lifespans. The pBNN analogues of the optimal models from each of the three data regimens yielded wide predictive uncertainties, reflecting the combined effects of aleatoric uncertainty from the activity retention parameters of Regimen 2 and the high epistemic uncertainty of Regimens 1 and 3.

The presented framework establishes the first systematic demonstration of applying contemporary ML to archival internal dosimetry datasets, defining the foundational data structure, temporal resolution, and measurement fidelity required for model generalisability. The study demonstrates that future progress in internal dosimetry will depend on comprehensive, individualised datasets, expanded temporal sampling through bioassay, and targeted organ-specific activity measurements, some of which exist in archival ^90^Sr ingestion studies [36]. Such datasets would enable the development of physics-informed neural networks that couple biokinetic transport principles with data-driven learning, extending beyond the stochastic approximations of holistic retention models applied in this study to the ITRI ^90^Sr beagle data. Overall, these findings demonstrate the utility of integrated intake reconstruction regimens to support dose estimation in modeling internal contamination events and to improve fidelity in representing radionuclide intake profiles.

## Data Availability

The data used in the development of all presented models is publicly available in PDF format through the Northwestern University Radiobiology Archive, under the ITRI Annual Reports page, LMF-120, page 569–571 with respect to the PDF file, and pages 565–567 with respect to the original document page numbering: https://sites.northwestern.edu/nura/data/inhalation-toxicology-research-institute-data/itri-annual-reports/.

## Code availability statement

Access to the repository with the code used in this work can be granted for academic or research purposes upon reasonable request to the corresponding author and under appropriate confidentiality agreements. Requests will be evaluated on a case-by-case basis.

## References

[1] ICRP (2016). Occupational intakes of radionuclides: part 2. ICRP publication 134. Ann. ICRP.

[2] ICRP (2006). Human alimentary tract model of radiological protection. ICRP publication 100. Ann. ICRP.

[3] ICRP (2015). Occupational intakes of radionuclides: part 1. ICRP publication 130. Ann. ICRP.

[4] Spencer H, Kramer L, Norris C, Samachson J (1972). Certain aspects of radiostrontium metabolism in man.

[5] Hollriegl V, Li W B, Oeh U (2006). Human biokinetics of strontium—part II: final data evaluation of intestinal absorption and urinary excretion of strontium in human subjects after stable tracer administration. Radiat. Environ. Biophs..

[6] Carr T, Harrison G, Loutit J, Sutton A (1962). Movement of strontium in the human body. Br. Med. J..

[7] Mate-Kole E M, Dewji S A (2024). Mathematical complexities in radionuclide metabolic modelling: a review of ordinary differential equation kinetic solvers in biokintic modelling. J. Radiol. Prot..

[8] Thompson R C (1989). Life-span effects of ionizing radiation in the beagle dog: a summary account of four decades of research funded by the U.S. Department of Energy and its predecessor agencies. https://s3.amazonaws.com/janus-cloud2/www/dog_tissues/reports_broken/red_book.pdf.

[9] Zander A, Paunesku T, Woloschak G E (2019). Radiation databases and archive—examples and comparisons. Int. J. Radiat. Biol..

[10] ITRI (1987). LMF-120. Inhalation Toxicology Research Institute Annual Reports.

[11] Woloschak Lab (2025). ITRI Annual Reports.

[12] ITRI (1966). LF-33. Inhalation Toxicology Research Institute Annual Reports.

[13] (2021). Fact Sheet: Inhalation Toxicology Laboratory, New Mexico, Site—an MED/AEC legacy site. Technical Report.

[14] ITRI (1968). LF-39. Inhalation Toxicology Research Institute Annual Reports.

[15] Boecker B, Aguilar F, Mercer T (1964). A canine inhalation exposure apparatus utilizing a whole body plethysmograph. Health Phys..

[16] Mate-Kole E M, Howard S C, Golden A P, Dewji S A (2024). Machine learning-enhanced stochastic uncertainty and sensitivity analysis of the ICRP human respiratory tract model for an inhaled radionuclide. J. Radiol. Prot..

[17] Kwon T-E, Chung Y, Yoo J, Ha W-H, Cho M (2020). Uncertainty quantification of bioassay functions for the internal dosimetry of radioiodine. J. Radiat. Res..

[18] Schofield P N, Kulka U, Tapio S, Grosche B (2018). Big data in radiation biology in epidemiology; an overview of the historical and contemporary landscape of data and biomaterial archives. Int. J. Radiat. Biol..

[19] Wilson C, Adams G G, Patel P, Windham K, Ennis C, Caffrey E (2024). A review of recent low-dose research and recommendation for moving forward. Health Phys..

[20] Rahimkhani P (2024). Deep neural network for solving stochastic biological systems. Iran. J. Sci..

[21] Weiss R, Karimijafarbigloo S, Roggenbuck D, Rodiger S (2022). Applications of neural networks in biomedical data analysis. Biomedicines.

[22] ITRI (1971). LF-45. Inhalation Toxicology Research Institute Annual Reports.

[23] Lipsztein J, Melo D, Oliveira C, Bertelli L, Ramalho A (1998). The Goiânia ^137^Cs accident—a review of the internal and cytogenic dosimetry. Radiat. Prot. Dosim..

[24] ITRI (1970). LF-44. Inhalation Toxicology Research Institute Annual Reports.

[25] Deist T M, Patti A, Wang Z, Krane D, Sorenson T, Craft D (2019). Simulation-assisted machine learning. Bioinformatics.

[26] Kang K, Belkhale S, Kahn G, Abbeel P, Levine S (2019). Generalization through simulation: integrating simulated and real data into deep reinforcement learning for vision-based autonomous flight.

[27] Salama K (2021). Probabilistic Bayesian neural networks. Keras Documentation.

[28] Kim Y, Kim M K, Fu N, Liu J, Wang J, Srebric J (2025). Investigating the impact of data normalization methods on predicting electricity consumption in a building using different artificial neural network models. Sustain. Cities Soc..

[29] Machine Learning in Plain English (2023). Deep learning course—lesson 7.4: ADAM (adaptive momement estimation). Medium.

[30] Fortmann-Roe S (2012). Understanding the bias-variance tradeoff. Essays by Scott Fortmann-Roe.

[31] Ranglani H (2025). Empirical analysis of the bias-variance tradeoff across machine learning models. Soc. Sci. Res. Netw. J..

[32] Deshpande S, Watson L T, Canfield R A (2013). Pareto front approximation using a hybrid approach. Procedia Comput. Sci..

[33] ITRI (1967). LF-38. Inhalation Toxicology Research Institute Annual Reports.

[34] Allodji R S, Leuraud K, Bernhard S, Henry S, Bénichou J, Laurier D (2012). Assessment of uncertainty associated with measuring exposure to radon and decay products in the French uranium miners cohort. J. Radiol. Prot..

[35] Kercher J R, Robison W L (1993). Uncertainties in predicted radionuclide body burdens and doses from discrete stochastic source terms. Health Phys..

[36] Glasco A D, Snyder L A, Paunesku T, Howard S C, Hooper D A, Golden A P, Woloschak G E (2024). Revisiting the historic strontium-90 ingestion beagle study conducted at the University of California Davis: opportunity in archival materials. Radiat. Res..

